# VIP-SPOT: an Innovative Assay To Quantify the Productive HIV-1 Reservoir in the Monitoring of Cure Strategies

**DOI:** 10.1128/mBio.00560-21

**Published:** 2021-06-22

**Authors:** Maria C. Puertas, Ángel Bayón-Gil, Maria C. Garcia-Guerrero, Maria Salgado, Víctor Urrea, Sara Morón-López, Ruth Peña, Esther Jiménez-Moyano, Bonaventura Clotet, Julia G. Prado, Javier Martinez-Picado

**Affiliations:** a AIDS Research Institute IrsiCaixa, Badalona, Spain; b University of Vic–Central University of Catalonia (UVic-UCC), Vic, Spain; c Germans Trias i Pujol Research Institute (IGTP), Badalona, Spain; d Catalan Institution for Research and Advanced Studies (ICREA), Barcelona, Spain; University of Utah School of Medicine; Albert Einstein College of Medicine

**Keywords:** HIV-1, HIV-1 cure, HIV-1 latent reservoir, HIV-1 reservoir size

## Abstract

Improved assays are critical to the successful implementation of novel HIV-1 cure strategies, given the limited ability of currently available assays to quantify true effects on the viral reservoir. As interventions based on immune clearance target infected cells producing viral antigens, irrespective of whether the viruses generated are infectious or not, we developed a novel assay to identify viral protein production at the single-cell level. The novel viral protein spot (VIP-SPOT) assay, based on the enzyme-linked ImmunoSpot (ELISpot) approach, quantifies the frequency of CD4^+^ T cells that produce HIV antigen upon stimulation. The performance of the VIP-SPOT assay was validated in samples from viremic (*n* = 18) and antiretroviral therapy (ART)-treated subjects (*n* = 35), and the results were compared with total and intact proviral DNA and plasma viremia. The size of the functional reservoir, measured by VIP-SPOT, correlates with total HIV-1 DNA and, more strongly, with intact proviruses. However, the frequency of HIV antigen-producing cells is 100-fold lower than that of intact proviruses, thus suggesting that most latently infected cells harboring full-length proviruses are not prone to reactivation. Furthermore, VIP-SPOT was useful for evaluating the efficacy of latency reversing agents (LRAs) in primary cells. VIP-SPOT is a novel tool for measuring the size of the functional HIV-1 reservoir in a rapid, sensitive, and precise manner. It might benefit the evaluation of cure strategies based on immune clearance, as these will specifically target this minor fraction of the viral reservoir, and might assist in the identification of novel therapeutic candidates that modulate viral latency.

## INTRODUCTION

In recent years, many efforts in the HIV-1 field have been directed toward finding a cure (1). HIV-1 cure would eliminate the drug-related toxicities associated with long-term antiretroviral therapy (ART), the risk of emergence of resistance mutations and/or transmission due to low adherence to chronic treatment, and the cost burden for the public health system. While complete eradication of the virus from the body remains unlikely, many novel therapeutic strategies have been designed to achieve a functional cure, which is defined as the ability of the individual to control the infection in the absence of ART. These strategies will probably need to reduce the viral reservoir and enhance immune responses in order to control viral burden. In the last decade, several clinical trials have tested the ability of latency reversing agents (LRAs) to purge the HIV-1 reservoir. Many LRAs (valproic acid, vorinostat, panobinostat, and romidepsin) are histone deacetylase inhibitors, which enhance gene transcription. The hypothesis underlying the use of LRAs is to “kick and kill” the latently infected cells by first reactivating viral expression, thus rendering them susceptible to cell death driven by a viral cytopathic effect and/or the immune system. The potential of some LRAs to disrupt HIV-1 latency *in vivo* has been demonstrated, although no significant impact on reducing the viral reservoir or, eventually, on viral control has been proven to date. For this reason, current clinical interventions aim to combine LRAs with immune stimulators such as HIV-1 therapeutic vaccines, broadly neutralizing antibodies, immune checkpoint inhibitors, and Toll-like receptor (TLR) agonists in order to boost the immune response, which could in turn target and eliminate reactivated HIV-1-infected cells (2).

One of the major drawbacks to the successful implementation and evaluation of these novel therapeutic interventions is our limited ability to quantify their true effect on the viral reservoir with a sensitive and precise *ex vivo* assay (3). The various available methods for measuring the viral reservoir have both strengths and weaknesses (4). The most sensitive and feasible way to determine the frequency of latently infected cells in antiretroviral therapy (ART)-treated patients is to quantify proviral HIV DNA in peripheral CD4^+^ T cells from blood samples by quantitative PCR. However, it has been shown that proviral quantification overestimates the size of the so-called “true” reservoir, since most integrated viral genomes are defective as a consequence of the high mutation and recombination rates of viral reverse transcriptase (5, 6). In addition, the proportion of defective proviruses varies widely between individuals. To overcome this issue, the recently developed intact proviral DNA assay (IPDA) uses multiplex droplet digital PCR (ddPCR) to infer the proportion of intact proviruses in a sample (7). The IPDA enables high-throughput analysis, but it assumes a proportion of misclassified defective sequences, which might be better identified by the alternative quadruplex quantitative PCR (Q4PCR) assay (8). The latter, however, is a labor-intensive method, and both assays are subject to probe amplification failures (9).

The only validated method for specifically quantifying the replication-competent viral reservoir is the quantitative viral outgrowth assay (qVOA) (10), which measures the number of cells containing viruses able to produce new rounds of infection upon *in vitro* stimulation. The time and costs of this methodology, added to the fact that a large number of cells are needed to perform the technique, make it impractical for regular use as a primary endpoint in most clinical trials. Although historically considered the gold standard assay for measuring the true reservoir, recent sequencing analyses have revealed that only 1 to 4% of full-length, intact proviruses are actually detected in the qVOA (5, 6, 11). To overcome this limitation, several methods have recently been developed to quantify the frequency of inducible proviruses (3, 12). These can measure the number of cells with the ability to produce either HIV-1 RNA (cell associated or in culture supernatant) or viral protein (Simoa technology; Quanterix, Inc.) (13–18) and are more sensitive and faster than the “classic” qVOA. Still, they all rely on a binomial positive/negative readout, thus implying the need for a limiting-dilution culture format to calculate the size of the inducible reservoir by maximum-likelihood statistical methods. Recent assays based on flow cytometry characterize the translation-competent viral reservoirs either by combining fluorescent *in situ* RNA hybridization and intracellular staining (19) or by duplex intracellular staining (20). However, the so-called HIV-Flow methodologies may be hampered by the high unspecific signal of anti-p24 antibodies routinely used for flow cytometry.

Hence, it is necessary to find rapid and scalable assays that enable the identification of cells with the ability to reverse HIV-1 from latency and to produce viral antigens, which are the target of immune-based therapies. To address this challenge, we aimed to develop a sensitive and high-throughput assay to quantify the productive viral reservoir at the single-cell level. We decided to set up an adapted enzyme-linked ImmunoSpot (ELISpot) protocol based on its ability to quantify individual cells expressing viral proteins in the culture wells and considering the high sensitivity of this kind of assays for the analysis of rare cell populations.

## RESULTS

### VIP-SPOT assay detects HIV-1 protein production at the single-cell level.

**FIG 1:**
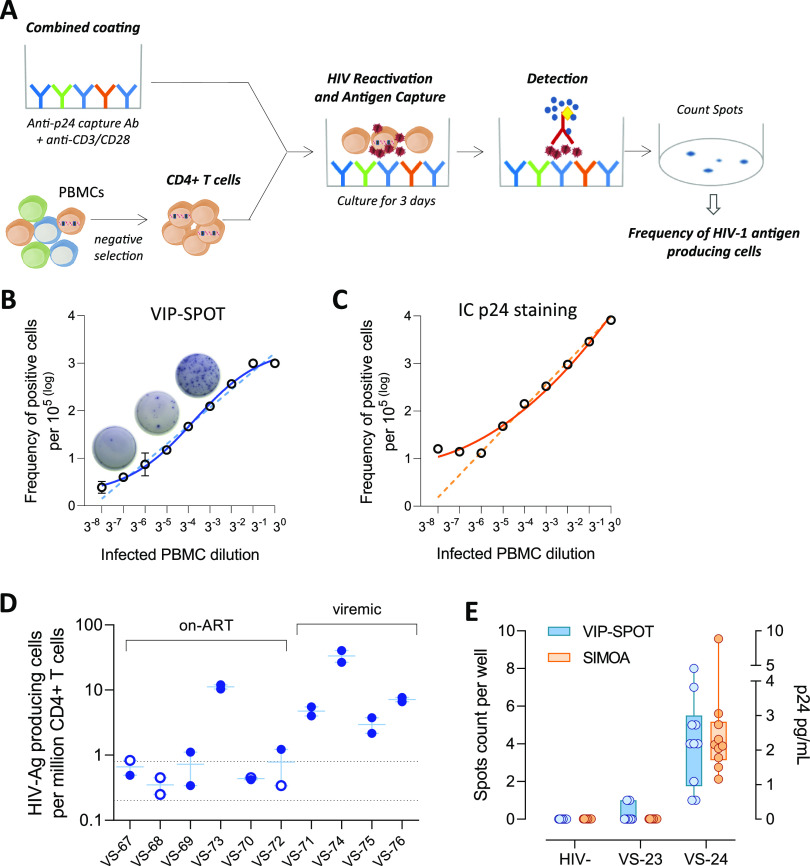
Viral protein spot (VIP-SPOT) assay detection of HIV-1 p24 production at the single-cell level. (A) Outline of the VIP-SPOT assay: measurement of the frequency of HIV-1 antigen-producing cells in primary samples from HIV-positive individuals. Enzyme-linked ImmunoSpot (ELISpot) plates were coated using a combination of antibodies (Ab) for p24 capture and anti-CD3/anti-CD28 for cell activation. CD4^+^ T cells were isolated from peripheral blood mononuclear cells (PBMCs) and cultured at 300,000 cells per well in the plates for 3 days before p24 detection. The frequency of HIV antigen-producing cells was calculated from the spot count (sum of all wells) and normalized by total cell input. (B and C) The sensitivity of VIP-SPOT was evaluated by comparing the frequency of HIV antigen-producing cells measured using VIP-SPOT and intracellular p24 staining using the KC57 antibody and flow cytometry analysis. PBMCs infected *in vitro* with HIV-1_NL4-3_ were spiked at different ratios (undiluted and 8 3-fold serial dilutions) in uninfected PBMCs from the same donors and tested in parallel using both assays. Predicted linear regression is represented as a dashed line, and nonlinear fit curves from observed data are shown as solid lines. (D) Repeated VIP-SPOT measures in two independent experiments (*n* = 10). Nondetection of HIV antigen-producing cells is represented as open symbols. The dashed line indicates the range of the limit of detection, which depended on the cell input in each sample. Six samples (VS-67, VS-68, VS-69, VS-70, VS-72, and VS-73) came from individuals on antiretroviral therapy (ART) and 4 came from viremic patients (VS-71, VS-74, VS-75, and VS-76). (E) Soluble p24 was analyzed using ultrasensitive digital enzyme-limited immunosorbent assay (ELISA) (Simoa platform) in supernatants from culture wells in the VIP-SPOT assay. Two samples from individuals with untreated HIV-1 infection (VS-23 and VS-24) and a sample from one noninfected donor as control (HIV-negative [HIV^−^]) were included in this assay. Ten replicates (culture wells) from each sample were tested.

We developed the viral protein spot (VIP-SPOT) assay, a method to detect HIV-1 p24 production at the single-cell level. To adapt the classic ELISpot assay to measure the productive reservoir in primary samples from individuals with HIV-1 infection, we formulated a combined coating of the plates to reactivate the latent reservoir using anti-CD3 and anti-CD28 antibodies and to simultaneously capture the antigen produced by the cells with an anti-p24 antibody (Fig. 1A).

To evaluate the linearity and sensitivity of the VIP-SPOT assay, we first tested serial dilutions of peripheral blood mononuclear cells (PBMCs) infected *in vitro* with HIV-1_NL4-3_ spiked in uninfected cells from the same donors. As a comparative analysis, we performed intracellular p24 staining and detection by flow cytometry (KC57 antibody). At higher concentrations of infected cells, intracellular staining performed better, as the ELISpot wells were easily saturated (Fig. 1B and C). It is of note that this would not affect the performance of VIP-SPOT in primary samples from HIV-infected individuals, as the frequency of HIV-1 infected cells is much lower than the range observed in infections *in vitro.* Conversely, for the lower dilutions tested, VIP-SPOT yielded better linearity results, probably due to the high background in KC57 staining.

Then, to assess the reproducibility of VIP-SPOT, we quantified the frequency of HIV antigen–producing cells in 10 samples from individuals with HIV-1 (4 viremic and 6 ART suppressed) (Fig. 1D). Two independent experiments were performed from cryopreserved PBMCs using 1 × 10^6^ to 3 × 10^6^ purified CD4^+^ T cells in each experiment, depending on cell recovery. The mean coefficient of variation was 0.32, indicating that the VIP-SPOT assay is reproducible. Slightly increased variability was observed in some samples, with results close to the limit of detection of the assay, indicating that a larger number of input cells would increase the robustness of the assay in those cases.

To assess the capacity of VIP-SPOT for detection of HIV-1 protein production at the single-cell level, we made a comparison with the HIV-Flow assay and with the digital enzyme-linked immunosorbent assay (ELISA). The experiments were performed in parallel in 2 samples from individuals with untreated HIV-1 infection and an uninfected donor as a control. The HIV-Flow assay, based on the use of duplex anti-p24 intracellular staining, did not detect p24 production in the samples tested, even though a clear double-positive signal was observed in positive controls run in parallel (PBMCs infected *in vitro* with HIV-1_NL4-3_ and J-Lat cell line) (see Fig. S1 in the supplemental material). Further studies with a larger number of samples would be needed to obtain conclusive results regarding this discordance between assays. For the ultrasensitive digital ELISA, we collected supernatants from the VIP-SPOT cultures (10 wells from each sample) just before washing the plates for subsequent detection steps and used them for ultrasensitive p24 measurement on the Simoa platform (Quanterix, Inc.). As shown in Fig. 1E, soluble p24, mostly at levels below 4 pg/ml, was effectively detected in the supernatants from the VS-24 culture wells, but not in those from VS-23 or uninfected donor wells, in agreement with data from the VIP-SPOT assay. The level of soluble p24 detected in the supernatants did not correlate significantly with the number of spots detected in each individual well, although a positive trend was observed (see Fig. S2 in the supplemental material). This may be due to low potency of the test because few samples were analyzed, or it might be the result of the variation in the amount of viral protein production and secretion of each infected cell, consistent with previous observations by Passaes et al. (16).

10.1128/mBio.00560-21.1 FIG S1 HIV-Flow. Primary CD4^+^ T cells from 2 viremic patients and 1 HIV-infected donor were analyzed in parallel using viral protein spot (VIP-SPOT) (Fig. 1E) and HIV-Flow methods for experimental comparison. Data from the flow cytometry analysis of the HIV-Flow assay (KC54-RD1 and 28B7-APC duplex staining) are shown. Dot blots are shown for the negative control (CD4^+^ T cells from noninfected controls) (A), cells from HIV-1-infected patients (B and C), and positive controls, namely *in vitro* HIV-infected CD4^+^ T cells (D) and J-Lat cells (E and F). Download FIG S1, EPS file, 1.7 MB. Copyright © 2021 Puertas et al. 2021 Puertas et al. https://creativecommons.org/licenses/by/4.0/ This content is distributed under the terms of the Creative Commons Attribution 4.0 International license.

10.1128/mBio.00560-21.2 FIG S2 Correlation between the number of spots detected by the VIP-SPOT assay and the level of p24 detected in the supernatants from the same wells by ultrasensitive digital enzyme-limited immunosorbent assay (ELISA) (sample VS-24 only). Result from the Spearman rank correlation test is shown. Download FIG S2, EPS file, 1.1 MB. Copyright © 2021 Puertas et al. 2021 Puertas et al. https://creativecommons.org/licenses/by/4.0/ This content is distributed under the terms of the Creative Commons Attribution 4.0 International license.

### VIP-SPOT correlates with other measures of viral burden in untreated HIV-1 infection.

**FIG 2:**
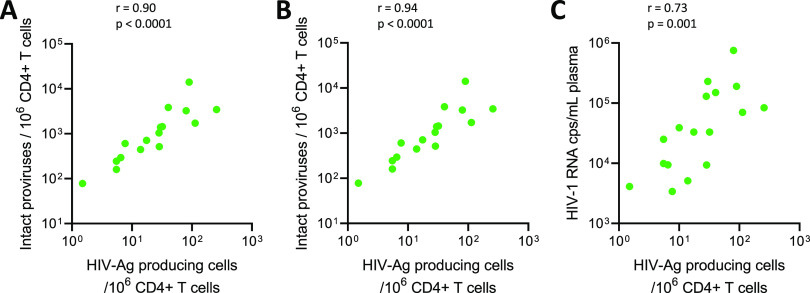
Correlation between VIP-SPOT and other HIV-1 measures of viral burden in viremic samples. Spearman’s rank correlation test was used for statistical analysis (*n* = 18). (A) Correlation between the frequency of HIV antigen (Ag)-producing CD4^+^ T cells in peripheral blood and total HIV-1 DNA, as measured by droplet digital PCR (ddPCR). (B) Correlation between the frequency of HIV antigen-producing cells and intact proviruses. (C) Correlation between VIP-SPOT data and plasma viral load.

To characterize the relationship between the frequency of HIV antigen-producing cells measured by VIP-SPOT and other markers of viral burden during the natural course of infection, we performed side-by-side quantifications in PBMC samples from 18 untreated individuals (see Table S1 in the supplemental material). Besides plasma viral load, we also measured the overall frequency of infected cells through the quantification of total HIV DNA in CD4^+^ T cells by ddPCR. Furthermore, as it has been widely reported that a major fraction of integrated HIV-1 genomes contains large deletions and is therefore not productive, we also used the novel IPDA to infer the proportion of CD4^+^ T cells bearing the whole viral genome. We observed that the frequency of cells able to produce p24 in viremic samples correlated strongly with total HIV DNA and with the frequency of intact proviruses (*r* = 0.90 and 0.94, respectively; *P* < 0.0001 for both) (Fig. 2). Furthermore, VIP-SPOT results also correlated with plasma viral load (*r* = 0.73; *P* = 0.001) in untreated infection.

10.1128/mBio.00560-21.4 TABLE S1 Clinical characteristics of viremic individuals. Download Table S1, DOCX file, 0.02 MB. Copyright © 2021 Puertas et al. 2021 Puertas et al. https://creativecommons.org/licenses/by/4.0/ This content is distributed under the terms of the Creative Commons Attribution 4.0 International license.

### VIP-SPOT measures the inducible HIV antigen-producing reservoir and correlates with other measures of viral persistence during suppressive ART.

**FIG 3:**
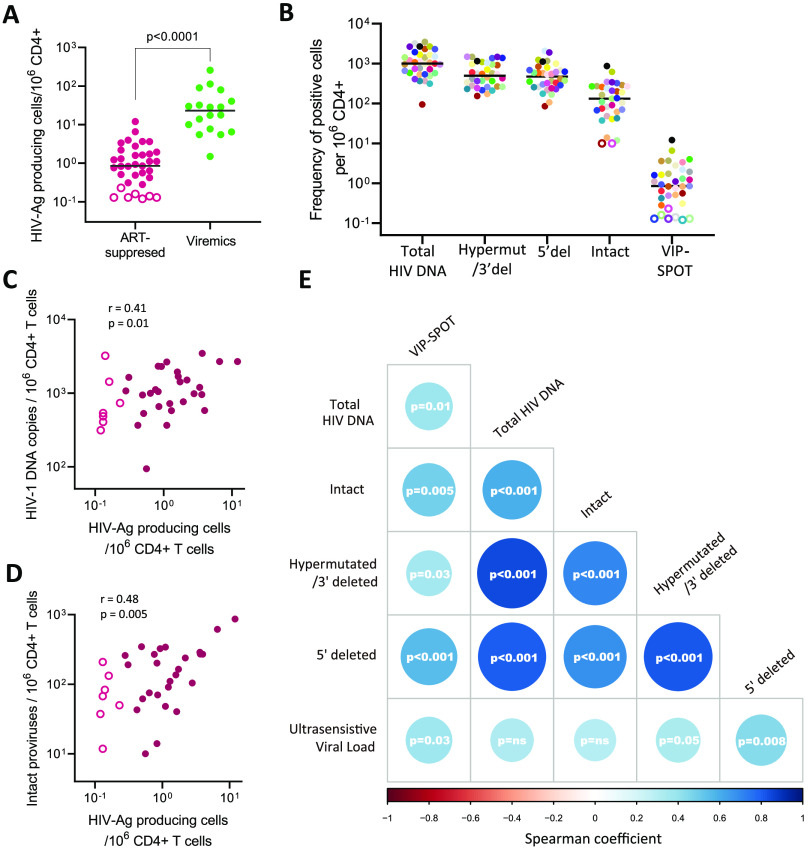
Correlation between VIP-SPOT and other assays measuring the size of the HIV-1 reservoir in samples from ART-suppressed individuals. (A) Comparative analysis of the frequency of HIV antigen producing-cells in samples from viremic and ART-suppressed individuals. Open symbols indicate samples in which no spots were detected. For representation and for correlation analysis, we used half of the limit of detection, calculated based on the total number of cells assayed from each sample. (B) Frequency of HIV antigen-producing cells was compared with total, defective, and intact HIV DNA from the same ART-suppressed individuals. (C and D) Correlation between the frequency of HIV antigen-producing CD4^+^ T cells in peripheral blood and total HIV-1 DNA and intact proviruses, respectively. (E) Correlation matrices between all of the different HIV-1 reservoir measures evaluated, based on the result of Spearman’s rank correlation tests (*n* = 35). The size and color intensity of the circles indicates the strength and direction of the correlation (blue indicates a positive correlation); *P* values for each comparison are indicated.

We used the VIP-SPOT assay to measure the frequency of inducible HIV antigen-producing cells in samples from 35 ART-treated subjects on viral suppression for at least 1 year (see Table S2 in the supplemental material). As expected, VIP-SPOT values in suppressed individuals were significantly lower than those observed in viremic subjects (Fig. 3A). In 7 of the 35 samples, we were not able to detect HIV antigen-producing cells, most probably because we were beyond the limit of detection of the assay in some cases. Here, a range of 1.4 × 10^6^ to 4 × 10^6^ purified CD4^+^ T cells from two vials of cryopreserved PBMCs were analyzed from each subject (see Fig. S3 in the supplemental material). Note that we intentionally avoided higher numbers of cells in this proof-of-concept analysis to evaluate the potential of VIP-SPOT in retrospective studies in which sample material is limited. However, starting with a larger number of cells or with fresh samples might improve the sensitivity of the assay in samples with the lowest levels of inducible reservoir.

10.1128/mBio.00560-21.3 FIG S3 Relationship between the total number of CD4^+^ T cells evaluated at each sample (on-ART individuals) and the frequency of antigen-producing cells detected. Download FIG S3, EPS file, 1.1 MB. Copyright © 2021 Puertas et al. 2021 Puertas et al. https://creativecommons.org/licenses/by/4.0/ This content is distributed under the terms of the Creative Commons Attribution 4.0 International license.

10.1128/mBio.00560-21.5 TABLE S2 Clinical characteristics of individuals on antiretroviral therapy (ART). Download Table S2, DOCX file, 0.02 MB. Copyright © 2021 Puertas et al. 2021 Puertas et al. https://creativecommons.org/licenses/by/4.0/ This content is distributed under the terms of the Creative Commons Attribution 4.0 International license.

Compared with other well-established reservoir measures, based on HIV DNA detection, we found the frequency of inducible p24-producing cells to be some 1,000-fold lower than total HIV DNA and 100-fold lower than the intact proviral reservoir (Fig. 3B). These results are in line with those of Pardons et al. and Ho et al. (5, 20), indicating that viral reactivation is strongly restrained in long-lived reservoir cells.

Even so, we found that the frequency of HIV antigen-producing cells detected by VIP-SPOT correlated positively with total HIV DNA (*r* = 0.41; *P* = 0.01) and, more strongly, with intact proviral levels (*r* = 0.48; *P* = 0.005) (Fig. 3C and D). These results suggest a close relationship between the reservoir susceptible of producing viral antigen upon stimulation and those cells containing full-length viral genomes, even though a complete overlap may not exist. Furthermore, a level of correlation was found between the VIP-SPOT results and low-level viremia (*r* = 0.36; *P* = 0.03) (Fig. 3E), while no association was found between the levels of ultrasensitive viral load and total or intact HIV DNA. This might indicate that the frequency of circulating HIV antigen-producing cells might be the origin, or at least a surrogate marker, of residual viremia under suppressive ART.

### VIP-SPOT reveals differential reservoir induction by LRAs in primary cells.

**FIG 4:**
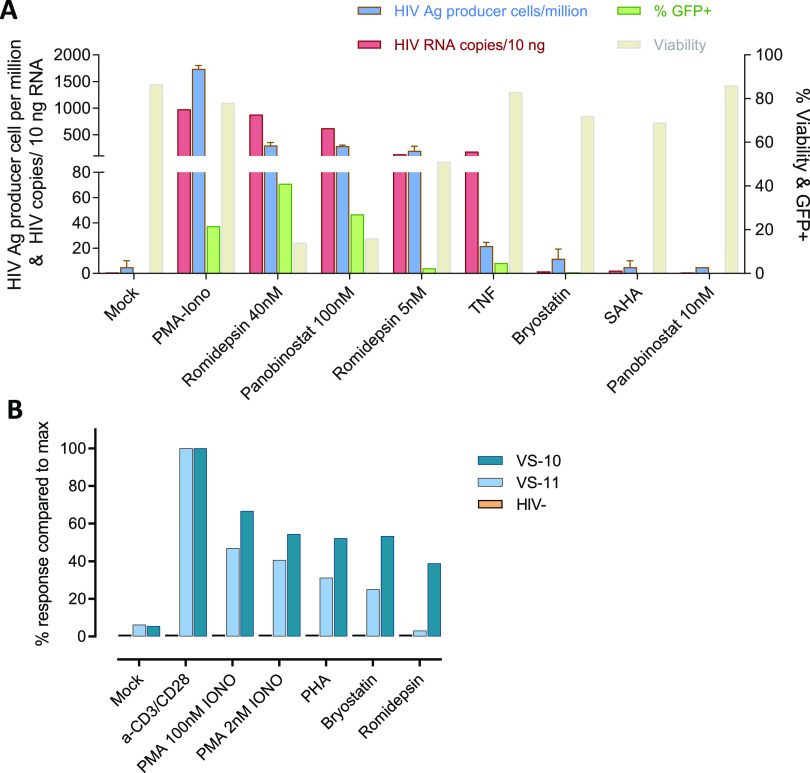
Latency reactivation testing using the VIP-SPOT assay. (A) Detection of reactivation of HIV-1 in J-Lat cells. Comparison of various latency reversing agents and the different readouts that can be used to detect the activity of the LRAs in this latency model, i.e., percentage of green fluorescent protein-positive (GFP^+^) cells and viability analyzed by flow cytometry, cell-associated HIV-1 RNA measured by ddPCR, and frequency of HIV antigen-producing cells detected by VIP-SPOT assay. (B) Comparison of the magnitude of the reactivation effect by different latency reversing agents (LRAs) according to HIV antigen production in primary CD4^+^ T cells measured by VIP-SPOT. Representative experiment included 2 samples from HIV-infected individuals and 1 from a noninfected (HIV^−^) donor.

To explore other potential applications of the VIP-SPOT assay beyond measurement of the reservoir, we tested the possibility of adapting the VIP-SPOT assay, using alternatives to coated anti-CD3/anti-CD28 as stimuli, to compare the efficiency of LRAs. First, we sought to determine the capacity of the VIP-SPOT assay to detect and quantify viral reactivation in the latently infected J-Lat cell line model (clone no. 8.4) and to compare it with other reactivation readouts. Thus, we also evaluated viral reactivation by measuring the proportion of green fluorescent protein (GFP)-positive cells by flow cytometry and quantifying cell-associated viral RNA by ddPCR. After culturing the cells with the different stimuli for 48 h, we were able to detect viral reactivation with tumor necrosis factor (TNF), phorbol myristate acetate (PMA)/ionomycin, panobinostat, and romidepsin (Fig. 4A). However, viral reactivation levels, as measured using the different assays, did not correlate in all cases. It is of note that J-Lat cells stimulated with TNF yielded high levels of viral mRNA, while low levels of GFP and p24 production were detected. These results support the notion that many different mechanisms may be involved in latency retainment beyond the transcription step, thus reinforcing the need to reevaluate the role of different LRAs if they have been previously assayed using technologies other than the one described here, based on detection of p24 production and secretion.

To further assess the ability of VIP-SPOT to test latency reversal in primary cells, we isolated CD4^+^ T cells from HIV-1-infected individuals and cultured them for 3 days in the presence of different activation stimuli (Fig. 4B). As expected, the VIP-SPOT results showed very limited detection of viral protein production in cells cultured without stimuli, while general activator stimuli (phytohemagglutinin [PHA], PMA/ionomycin, and, most notably, anti-CD3/anti-CD28 antibodies) resulted in enhanced detection of HIV-1 antigen-producing cells. It is of note that histone deacetylase inhibitors (HDACIs) were able to induce detectable latency reactivation in primary CD4^+^ T cells from infected individuals, and, contrary to the observations in J-Lat cells, this was more pronounced with protein kinase C (PKC) modulators (bryostatin) than with romidepsin. Despite the fact that a limited number of samples were tested here, our results reinforce the importance of implementing LRA testing platforms that include patient-derived CD4^+^ T cells in order to obtain reliable information about their potential utility.

## DISCUSSION

Here, we report data on the development and validation of VIP-SPOT, a novel methodology aimed at measuring the size of the functional HIV-1 reservoir in a rapid, sensitive, and precise manner. Based on ELISpot technology, this high-throughput assay enables the detection at the single-cell level of CD4^+^ T lymphocytes with the potential to produce viral proteins. We established this assay with the purpose of getting a novel biomarker to uncover the impact of novel therapeutic interventions precisely on the functional fraction of the viral reservoir.

The concept of using such an immune spot assay to detect and enumerate HIV-1 antigen-secreting cells has been assessed elsewhere (21), and the method was already used to measure latently infected CD4^+^ T cells from HIV-1-infected individuals (from peripheral blood and breast milk) (22, 23). However, the original protocol included the use of pooled sera from HIV-1-infected individuals with a complete HIV-1-specific serologic pattern in Western blots for plate coating. Here, we developed a novel protocol that obviates the need for human serum and uses monoclonal antibodies instead in order to reduce variability between experiments and increase sensitivity. Furthermore, we screened several combinations of coating and capture antibodies (see Table S3 in the supplemental material), as well as CD4^+^ T-cell culture stimuli, to improve the robustness and sensitivity of the assay.

10.1128/mBio.00560-21.6 TABLE S3 List of antibody pairs tested for coating and detection in the VIP-SPOT assay. The antibody pair that was finally selected and implemented in the final protocol, based on higher sensitivity against a panel of patient-derived viral isolates, is indicated in bold. Download Table S3, DOCX file, 0.02 MB. Copyright © 2021 Puertas et al. 2021 Puertas et al. https://creativecommons.org/licenses/by/4.0/ This content is distributed under the terms of the Creative Commons Attribution 4.0 International license.

Since the number of cells available from clinical trials can be limited, the VIP-SPOT assay was validated in the present study entirely with retrospective cryopreserved samples as proof of concept that small blood volume would be efficiently tested. This is a clear benefit of using a single-cell detection platform that is not based on a limiting-dilution format and qualitative readout, as in other widely used assays, such as qVOA and inducible RNA assays. Therefore, VIP-SPOT could also be used to measure the frequency of HIV antigen-producing cells from tissue samples, where target cell recovery may be an issue. Still, our results indicate that the sensitivity/reproducibility of the assay might be improved if a larger number of cells were interrogated, especially when the frequency of latently infected cells is extremely low. Because of the study design, the reduced sample availability hampered direct comparison of all validated assays for measurement of the HIV-1 reservoir, including qVOA, thus limiting the present analysis.

During the natural course of HIV infection, VIP-SPOT showed a strong correlation with DNA- and RNA-based assays that measure viral burden, thus confirming its robustness. Likewise, in ART-suppressed individuals, direct correlations, while somewhat weaker, were statistically significant. While it has been reported that defective viral genomes may produce Gag protein (24), the strongest correlation found here, between the frequency of positive cells detected by VIP-SPOT and IPDA, suggests that a large proportion of those antigen-producing cells contain an intact provirus. It is of note that previous studies in which cell-associated HIV-1 RNA was evaluated as a marker of inducible HIV-1 (*tat*/*rev*-induced limiting dilution assay [TILDA]) did not find this level of correlation with IPDA-intact proviruses (25). However, the mean frequency of HIV antigen-producing cells observed here is much lower than the number of intact proviruses in samples. These results are in line with previous observations by Ho et al., which demonstrated that most proviral genomes are not prone to latency reactivation during a single round of stimulation, despite being replication competent according to sequence integrity (5). Indeed, other groups have also reported evidence of a fraction of intact proviruses in latently infected cells that are not susceptible to CD8^+^ T-cell targeting upon latency reversal (26). This may indicate that some “intact” proviruses are in fact defective, integrated in chromosomal sites refractory to induction, or hosted by cells refractory to activation. Likewise, latency blockade downstream of the initial transcription steps (e.g., in viral RNA elongation and splicing, nuclear export, protein translation, and virion assembly/release) may limit the production of new viral particles and antigen presentation (27–29). In that context, *in vitro* and *in vivo* testing of LRAs by inducible RNA assays (14, 30)—used to measure unspliced viral RNA (vRNA) or multiple-spliced transcripts as surrogate markers—may overestimate the potency of some LRAs. Thus, the use of assays based on the detection of viral proteins—or viral particles—produced by reactivated cells may provide more reliable information. Another advantage of VIP-SPOT is that p24 capture on the well membrane is cumulative over the 3 days of culture. This enables the capture of all antigens produced during this period, and not only within a specific time frame, as is the case with other inducible HIV-1 assays based on intracellular staining, such as the combined mRNA Flow-fluorescent *in situ* hybridization (FISH) assay or HIV-Flow (19, 20). The opportunity to capture antigen production over a longer window period may benefit the identification of reactivation induced by LRAs with different kinetics of response. Conversely, a limitation of this assay is that no further phenotypic analysis from the detected HIV antigen-producing cells can be performed.

The VIP-SPOT assay quantifies the frequency of latently infected CD4^+^ T cells that produce viral protein upon stimulation. Although we are aware that this would be considered an overestimation of the “true” replication-competent viral reservoir, which is the final objective of cure strategies, we hypothesize that any therapeutic strategy based on latency reactivation and immune clearance will target all infected cells producing viral antigens, independently of whether they are capable of producing infectious virions or not. Therefore, if the main objective is to be able to evaluate the impact of these clinical interventions on the viral reservoir, it will be highly valuable and easier to detect this effect if HIV-1 antigen-producing cells are specifically estimated. Standard qVOA assays may not be sensitive enough to detect those changes, and measurement of total HIV DNA (proviral reservoir) would prevent the effect on the minor productive fraction from being observed. Indeed, when the whole proviral reservoir is measured, its defective (and therefore unproductive) fraction would be too large to enable detection of the impact on the minor but relevant antigen-producing subset, thus masking the effect of the therapeutic intervention under study.

In conclusion, VIP-SPOT is a new platform for quantification of the functional reservoir at the single-cell level. The assay represents an advance in our repertoire of validated measures of the HIV-1 reservoir, it is suitable for clinical studies and might be adapted also to a variety of other applications, such as *in vitro* LRA testing or characterization of reactivation blockade steps. The VIP-SPOT assay is robust, sensitive, scalable, and suitable for longitudinal and multicenter retrospective/prospective clinical trials. While the frequency of antigen-producing cells measured by VIP-SPOT correlates with other DNA-based reservoir measures, this assay could prove highly beneficial for evaluation of therapeutic strategies based on immune clearance, since these will specifically target this minor fraction of the viral reservoir.

## MATERIALS AND METHODS

### Participants and samples.

We based the study on retrospective samples from 18 viremic and 35 ART-suppressed individuals. The inclusion criteria for viremic subjects were viremia of >1,000 HIV-1 RNA copies/ml and absolute CD4^+^ T-cell counts greater than 500 cells/μl; the median viral load was 33,000 HIV-1 RNA copies/ml (range, 3,400 to 750,000 copies/ml). ART-suppressed individuals had sustained HIV RNA levels of <50 copies/ml for at least 1 year and presented a CD4^+^ T-cell count of >200 cells/μl. Blood samples were collected in the context of previous studies conducted at Hospital Universitari Germans Trias i Pujol (Badalona, Spain) and had been stored as part of the IrsiCaixa sample collection. Written informed consent for access to the samples for the present study was obtained from the participants. The study was approved by the Hospital Ethics Review Committee (approval number AC-19-274).

As negative controls, we used PBMCs isolated from the buffy coats of tested HIV-negative blood donors. The buffy coats were purchased from the Catalan Banc de Sang i Teixits (Barcelona, Spain).

### VIP-SPOT assay.

ELISpot plates (Immobilon-P polyvinylidene difluoride membrane, catalog no. MSIPS4W10; Millipore) were coated with 50 μl per well of a mixture of capture and stimulating antibodies as follows: 10 μg/ml of mouse monoclonal antibody to HIV p24 (clone 39/5.4A, catalog no. ab9071; Abcam) and 1 μg/ml of each of the stimulating antibodies targeting CD3 and CD28 (clone OKT3, catalog no. 16-0037-81, and clone CD28.2, catalog no. 16-0289-85; eBioScience). This combined coating was prepared in sterile phosphate-buffered saline (PBS) and incubated overnight at 4°C. On the day of cell culture, the wells were washed 3 times with 200 μl of sterile PBS and incubated with 100 μl of a blocking solution containing 5% bovine serum albumin (BSA) (MACS BSA stock solution, catalog no. 130-091-376; Miltenyi) in PBS, for at least 30 min at room temperature. Then, the wells were washed 5 times with 200 μl of sterile PBS and prepared with 50 μl of culture medium with 30 U/ml interleukin 2 (IL-2) (Proleukin, catalog no. 703892-4; Roche) until addition of the cells.

To measure the productive viral reservoir in HIV-infected subjects, CD4^+^ T cells were isolated from 20 × 10^6^ to 30 × 10^6^ cryopreserved PBMCs (2 vials per sample) by negative immunomagnetic separation using the autoMACS Pro separator (CD4^+^ T-cell isolation kit; Miltenyi) according to the manufacturer’s instructions and counted using the automated NucleoCounter NC-3000 (Chemometec, Denmark). Cell suspensions containing up to 2 × 10^6^/ml of purified CD4^+^ T cells were prepared in culture medium (RPMI 1640 containing 20% fetal bovine serum [FBS], 100 U/ml penicillin, and 100 μg/ml streptomycin; all from Invitrogen) and distributed at 50 μl per well, with the result that 3 × 10^5^ cells per well were seeded in the precoated ELISpot plates. No antiretrovirals were added to the culture, as preliminary data showed no differences in spot count in presence or absence of 200 nM maraviroc. In all VIP-SPOT assays, CD4^+^ T cells from HIV-negative individuals were included as negative controls. Likewise, all plates included 2 positive-control wells containing J-Lat cells (clone 8.4), at an approximate ratio of 10^2^ cells/well, in culture medium supplemented with 20 nM PMA and 0.5 μg/ml ionomycin (catalog no. P1585 and I3909; Sigma-Aldrich).

After 3 days of incubation at 37°C in a 5% CO_2_-humified atmosphere, the plates were washed 6 times with 200 μl of PBS per well (automated) and incubated for 3 h with 0.5 μg/ml of a monoclonal anti-p24 antibody conjugated with biotin (clone 8G9, catalog no. NBP2-41337; Novus Biologicals) at room temperature. After 6 PBS washes, detection was performed for 45 min at room temperature using a solution of streptavidin-alkaline phosphatase conjugate (catalog no. 3310-10; Mabtech) diluted at 1:1,000 in PBS. After further 6 washes, the BCIP/NBT-plus substrate (catalog no. 3650-10; Mabtech) was added, and the chromogenic reaction developed for 8 min. After aspiration of the substrate, plate wells were incubated for 10 min at room temperature with 200 μl of 0.05% Tween 20 in PBS to inactivate any remaining virus before final washing with running water. After drying, the spots were counted using an automated ELISpot reader unit (Cellular Technology Limited, Shaker Heights, OH). The frequency of HIV-1 antigen-producing cells was determined based on the total number of spots detected for each sample and the amount of CD4^+^ T cells cultured.

### *In vitro* HIV-1 infection of primary cells.

PBMCs from HIV-negative donors were infected *in vitro* with the HIV-1_NL4-3_ lab strain (31) to test the sensitivity of the VIP-SPOT assay. For each experiment, PBMCs from 2 donors were cultured for 2 days with 3 μg/ml PHA (Sigma-Aldrich) in the presence of 10 U/ml IL-2 (Proleukin; Roche). Then, activated PBMCs were mixed and inoculated at a multiplicity of infection (MOI) of 0.001 for 1 h. After washing, cultures were maintained for 3 days in culture medium supplemented with 10 U/ml IL-2 and were subsequently used in the VIP-SPOT assays.

### Single and duplex intracellular p24 staining (HIV-Flow).

Single intracellular p24 staining was used to detect productive HIV-1 in primary cells infected *in vitro*. Three days after infection, serial one-third dilutions from the infected cultures were spiked into noninfected PBMCs from the same donors. Then, cells were fixed and permeabilized using the Fix & Perm kit (Invitrogen, previously Caltag) following the manufacturer’s instructions. During permeabilization, the RD1-stained anti-p24 monoclonal antibody (clone KC57; Beckton Dickinson) was added at a 1/50 dilution.

To detect p24-producing cells in samples from HIV-infected individuals, we performed the HIV-Flow protocol based on duplex intracellular staining, as previously described (20). Briefly, isolated CD4^+^ T cells were cultured at 2 × 10^6^ cells/ml in 24-well plates in the presence of 25 nM PMA and 1 μg/ml ionomycin (Sigma-Aldrich) for 24 h. Then, cells were stained for viability (Live/Dead fixable green dead cell stain kit; Molecular Probes) according to the manufacturer’s instructions before proceeding with the fixation and permeabilization protocol described above. Here, the anti-p24 antibodies KC57-RD1 (Beckton Dickinson) and 28B7-APC (MédiMabs) were both used at a 1/250 dilution for the double stain based on in-house titration using *in vitro*-infected PBMCs, as recommended. J-Lat cells (clone 8.4) were cultured and stained in parallel as positive controls. The flow cytometer FACScalibur (BD) and CellQuest Pro software (BD) were used for analysis.

### Ultrasensitive HIV-1 p24 digital immunoassay.

Viral p24 antigen in the supernatants was quantified using a single-molecule assay (SR-X Simoa analyzer; Quanterix). From each VIP-SPOT well, 119 μl of supernatant was collected immediately before plate development and lysed in 0.5% Triton X-100 (Sigma-Aldrich) before p24 determination. Using the Simoa HIV p24 advantage kit according to the manufacturer’s instructions, a calibration curve ranging from 0.006 to 28.7 pg/ml was established.

### Quantification of total and intact HIV DNA.

The proportion of CD4^+^ T cells harboring total HIV DNA and intact provirus was measured from purified CD4^+^ T cells using ddPCR, as previously described (7, 32). Briefly, for total HIV DNA, lysed extracts from isolated CD4^+^ T cells were used to quantify the frequency of proviral DNA based on primer/probe sets annealing at the 5′ long terminal repeat (LTR) or GAG, and the RPP30 cellular gene was measured in parallel to normalize sample input. To measure the frequency of intact provirus, duplex ddPCR was performed using the packaging signal (ψ) and nonhypermutated Env primer/probe sets, per the original IPDA protocol (7). For those samples failing Env detection, a secondary prime/probe set targeting Env was used to rescue intact provirus quantification (33). To normalize data and to correct for DNA shearing in each sample, 2 primer/probe sets targeting the RPP30 gene were used in a duplex ddPCR simultaneously run (33). All probes were double-quenched 6-carboxyfluorescein (FAM)/6-carboxy-2,4,4,5,7,7-hexachlorofluorescein (HEX)-ZEN-Iowa Black FQ and were purchased from IDT (Integrated DNA Technologies, Belgium). The results were analyzed using the QX100 droplet reader and QuantaSoft software version 1.6.6.0320 (Bio-Rad).

### Ultrasensitive plasma viral load (usVL).

Residual viremia had been measured in the contemporaneous plasma samples from ART-suppressed individuals in the context of a previous clinical trial (34). Briefly, up to 7.5 ml of plasma was ultracentrifuged prior to extraction and quantification of viral RNA using the m2000sp Abbott RealTime HIV-1 assay device and an in-house calibration curve set (range, 10^1^ to 10^3^ copies/ml).

### Latency reactivation assays.

J-Lat full-length cells (clone 8.4, NIH AIDS Reagent Program catalog no. 9847) were used as a model for latent HIV infection (35). For the VIP-SPOT assay, 10^4^ J-Lat cells per well were seeded in plates precoated with 10 μg/ml of the anti-p24 capture antibody (clone 39/5.4A, catalog no. ab9071; Abcam) and cultured for 48 h in the presence of different latency reversing agents (LRAs) prepared in culture medium, namely, 20 nM PMA plus 0.5 μg/ml ionomycin (catalog no. P1585 and I3909; Sigma-Aldrich), 10 ng/ml TNF (CellGenix), 1 μM vorinostat (SAHA), 10 nM and 100 nM panobinostat, 5 nM and 40 nM romidepsin, and 100 nM bryostatin (all from Santa Cruz Biotechnologies). Cells cultured under the same stimulus conditions were prepared in parallel in 48-well plates at a ratio of 10^6^ cells per well for further cytometry analysis of GFP expression and viability and for determination of cell-associated HIV RNA, as previously described (36). Briefly, one-step reverse transcription ddPCR (Bio-Rad) was used to quantify HIV RNA with a primer/probe set targeting the viral 5′ long terminal repeat.

For primary cells, latency reversing agents that were alternative to coated anti-CD3/anti-CD28 in the VIP-SPOT assay were tested as follows: 10 μg/ml PHA (L1668; Sigma-Aldrich), PMA at 2 nM or 100 nM combined with 0.5 μM ionomycin, 5 nM romidepsin, and 100 nM bryostatin.

### Statistical analysis.

All statistical analyses were performed using GraphPad Prism (version 8.4.3). The nonparametric Mann-Whitney test was used to compare results between groups. Spearman’s rank test was used to evaluate the correlation between different reservoir measures. The correlation matrix between the different HIV-1 reservoir measures was created using the *corrplot* package in R software.
