# Erratum for Puertas et al., “VIP-SPOT: an Innovative Assay To Quantify the Productive HIV-1 Reservoir in the Monitoring of Cure Strategies”

**DOI:** 10.1128/mBio.02535-21

**Published:** 2021-09-28

**Authors:** Maria C. Puertas, Ángel Bayón-Gil, Maria C. Garcia-Guerrero, Maria Salgado, Víctor Urrea, Sara Morón-López, Ruth Peña, Esther Jiménez-Moyano, Bonaventura Clotet, Julia G. Prado, Javier Martinez-Picado

**Affiliations:** a AIDS Research Institute IrsiCaixa, Badalona, Spain; b University of Vic–Central University of Catalonia (UVic-UCC), Vic, Spain; c Germans Trias i Pujol Research Institute (IGTP), Badalona, Spain; d Catalan Institution for Research and Advanced Studies (ICREA), Barcelona, Spain

## ERRATUM

Volume 12, no. 3, e00560-21, 2021, https://doi.org/10.1128/mBio.00560-21. Erratum for Fig. 2.

**Figure:**
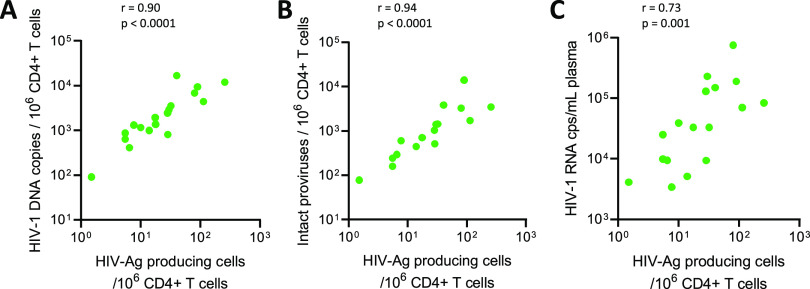


In Fig. 2, the images in panel A and B were duplicated by mistake. The correct Fig. 2A and B images appear below. There are no corresponding changes to the text or figure legend.

