# Supplementary material for: VIP-SPOT: an Innovative Assay To Quantify the Productive HIV-1 Reservoir in the Monitoring of Cure Strategies
Source: mBio. 2021 Jun 22;12(3):e00560-21. doi: 10.1128/mBio.00560-21 (PMC8262951; doi:10.1128/mBio.00560-21)
Supplement: TABLE S1 [file mbio.00560-21-st001.docx]

**Supplementary Table 1.**

| **Viremics** | | | |
| --- | --- | --- | --- |
| **Sample Code** | **Weeks from diagnostic** | **CD4 abs at sampling** | **Viral Load**  **at Sampling *** |
| VS-8 | 3.3 | 1097 | 230,000 |
| VS-9 | 6.7 | 956 | 39,000 |
| VS-10 | 21.1 | 1055 | 190,000 |
| VS-11 | 2.6 | 748 | 33,000 |
| VS-12 | 2.0 | 623 | 750,000 |
| VS-13 | 0.3 | 646 | 84,000 |
| VS-21 | 23.0 | 671 | 33,000 |
| VS-22 | 27.6 | 853 | 9,400 |
| VS-23 | 4.3 | 1062 | 4,100 |
| VS-24 | 3.3 | 586 | 130,000 |
| VS-30 | 1.3 | 530 | 9,343 |
| VS-36 | 4.6 | 565 | 5,100 |
| VS-48 | 0.0 | 652 | 70,000 |
| VS-54 | 0.7 | 596 | 25,000 |
| VS-71 | 0.0 | 725 | 9,933 |
| VS-72 | 4.1 | 1172 | 33,000 |
| VS-74 | 1.1 | 932 | 150,000 |
| VS-76 | 4.4 | 647 | 3,400 |
| **Median** | **3.3** | **698** | **3.3x10^4^** |
| [IQR] | [1-5.1] | [616-981] | [9.4x10^3^-1.3x10^5^] |

*VL at sampling in viremic individuals is the only clinical parameter correlating with the frequency of Ag-producing cells, intact provirus and total proviral levels (p-values=0.001, =0.001 and <0.001, respectively).
