# Supplementary material for: VIP-SPOT: an Innovative Assay To Quantify the Productive HIV-1 Reservoir in the Monitoring of Cure Strategies
Source: mBio. 2021 Jun 22;12(3):e00560-21. doi: 10.1128/mBio.00560-21 (PMC8262951; doi:10.1128/mBio.00560-21)
Supplement: TABLE S2 [file mbio.00560-21-st002.docx]

**Supplementary Table 2.**

| **Suppressed** | | | | | |
| --- | --- | --- | --- | --- | --- |
| **Sample Code** | **Years from diagnostic** | **CD4 nadir** | **VL zenit** | **CD4s at sampling** | **Years suppressed** |
| VS-25 | 32 | 200 | 369,999 | 1,770 | 6 |
| VS-26 | 11 | 509 | 89,991 | 1,130 | 3 |
| VS-27 | 11 | 247 | 359,998 | 730 | 5 |
| VS-28 | 30 | 614 | 75 | 1,610 | 1 |
| VS-29 | 33 | 66 | 1,270 | 940 | 13 |
| VS-31 | 10 | 211 | 8,500 | 620 | 1 |
| VS-32 | 29 | 247 | 630 | 1,110 | 4 |
| VS-33 | 29 | 192 | 369,999 | 1,280 | 4 |
| VS-34 | 6 | 398 | 139,991 | 930 | 4 |
| VS-35 | 25 | 30 | 2,200,000 | 610 | 13 |
| VS-37 | 18 | 14 | 14,000 | 810 | 6 |
| VS-38 | 14 | 206 | 9,400 | 350 | 1 |
| VS-39 | 12 | 121 | 165,006 | 900 | 4 |
| VS-40 | 17 | 399 | 350 | 1,240 | 4 |
| VS-41 | 20 | 6 | 78,000 | 1,180 | 10 |
| VS-43 | 22 | 191 | 51 | 710 | 5 |
| VS-44 | 18 | 30 | 2,000 | 730 | 1 |
| VS-45 | 22 | 100 | 160,000 | 1,550 | 13 |
| VS-46 | 15 | 45 | 4,400 | 460 | 9 |
| VS-47 | 26 | 124 | 270,000 | 470 | 10 |
| VS-49 | 20 | 20 | 120,000 | 610 | 10 |
| VS-50 | 19 | 117 | n.d. | 370 | 13 |
| VS-51 | 27 | 193 | 80,000 | 660 | 9 |
| VS-52 | 16 | 228 | 70,000 | 1,020 | 1 |
| VS-53 | 18 | 5 | 470,000 | 680 | 12 |
| VS-61 | 13 | 197 | 180,011 | 490 | 5 |
| VS-62 | 22 | 256 | 1,200,000 | 530 | 5 |
| VS-63 | 8 | 300 | 40,000 | 1,410 | 1 |
| VS-64 | 28 | 210 | 1,300,000 | 410 | 4 |
| VS-67 | 6 | 181 | 210,000 | 390 | 5 |
| VS-68 | 8 | 287 | 170,000 | 911 | 7 |
| VS-69 | 8 | 16 | 693,333 | 685 | 10 |
| VS-70 | 15 | 239 | 11,474 | 542 | 12 |
| VS-72b | 18 | 376 | 220,000 | 726 | 7 |
| VS-73b | 22 | 100 | 160,000 | 1,290 | 13 |
| **Median** | **18** | **193** | **1.3x10^5^** | **730** | **5** |
| [IQR] | [12-25] | [66-247] | [9.2x10^3^-2.9x10^5^] | [542-1130] | [4-10] |

* None of these clinical parameters correlate with the frequency of Ag-producing cells, intact provirus or total HIV-1 proviral levels.
