# Supplementary material for: VIP-SPOT: an Innovative Assay To Quantify the Productive HIV-1 Reservoir in the Monitoring of Cure Strategies
Source: mBio. 2021 Jun 22;12(3):e00560-21. doi: 10.1128/mBio.00560-21 (PMC8262951; doi:10.1128/mBio.00560-21)
Supplement: TABLE S3 [file mbio.00560-21-st003.docx]

**Supplementary Table 3.**

| **Antibody pairs tested for VIP-SPOT** | |
| --- | --- |
| **p24 Coating antibody** | **p24 Detection antibody** |
| MoAb 39/6.14 (ab9072, abcam) | ​Goat *PolyAb* ab20774 with biotin (abcam) |
| MoAb 39/6.14 (ab9072, abcam) | Goat *PolyAb* ab68617 with biotin (abcam) |
| MoAb 39/5.4A (ab9071, abcam)​ | Goat *PolyAb* ab20774 with biotin (abcam) |
| MoAb 39/5.4A (ab9071, abcam)​ | Goat *PolyAb* ab68617 with biotin (abcam) |
| MoAb [7F4] (Sigma, SAB3500882) | Goat *PolyAb* ab20774 with biotin (abcam) |
| MoAb [7F4] (Sigma, SAB3500882) | Goat *PolyAb* ab68617 with biotin (abcam) |
| MoAb [7F4] (Sigma, SAB3500882) | MoAb [8G9]​ with biotin (NBP2-4133, Novus) |
| **MoAb 39/5.4A (ab9071, abcam)​** | **MoAb [8G9]​ with biotin (NBP2-4133, Novus)** |
| MoAb 39/6.14 (ab9072, abcam) | MoAb [8G9]​ with biotin (NBP2-4133, Novus) |
| MoAb [24-2] (6457, NIH AIDS Reagent Program) | MoAb [8G9]​ with biotin (NBP2-4133, Novus) |
| MoAb N29 (ab63959, abcam) | MoAb [8G9]​ with biotin (NBP2-4133, Novus) |
| MoAb [7F4] (Sigma, SAB3500882) | MoAb 39/5.4A (ab9071)​ in-house biotilinated |
| MoAb 39/6.14 (ab9072, abcam) | MoAb 39/5.4A (ab9071)​ in-house biotilinated |
| MoAb [24-2] (6457, NIH AIDS Reagent Program) | MoAb 39/5.4A (ab9071)​ in-house biotilinated |
| MoAb N29 (ab63959, abcam) | MoAb 39/5.4A (ab9071)​ in-house biotilinated |
| MoAb 39/5.4A (ab9071, abcam)​ | MoAb 39/6.14 (ab9072)​ in-house biotilinated |
| MoAb [7F4] (Sigma, SAB3500882) | MoAb 39/6.14 (ab9072)​ in-house biotilinated |
| MoAb [24-2] (6457, NIH AIDS Reagent Program) | MoAb 39/6.14 (ab9072)​ in-house biotilinated |
| MoAb N29 (ab63959, abcam) | MoAb 39/6.14 (ab9072)​ in-house biotilinated |
| MoAb 39/5.4A (ab9071, abcam)​ | MoAb N29 (ab63959) in-house biotinilated |
| MoAb 39/6.14 (ab9072, abcam) | MoAb N29 (ab63959) in-house biotinilated |
| MoAb [7F4] (Sigma, SAB3500882) | MoAb N29 (ab63959) in-house biotinilated |
